# An improved SYBR Green-1-based fluorescence method for the routine monitoring of *Plasmodium falciparum* resistance to anti-malarial drugs

**DOI:** 10.1186/s12936-015-1011-x

**Published:** 2015-12-01

**Authors:** Victor Dery, Nancy O. Duah, Ruth Ayanful-Torgby, Sena A. Matrevi, Francis Anto, Neils B. Quashie

**Affiliations:** Department of Animal Biology and Conservation Sciences, University of Ghana, Accra, Ghana; Epidemiology Department, Noguchi Memorial Institute for Medical Research, University of Ghana, Accra, Ghana; Centre for Tropical Clinical Pharmacology and Therapeutics, School of Medicine and Dentistry, University of Ghana, Accra, Ghana; School of Public Health, University of Ghana, Accra, Ghana

**Keywords:** Fluorescence, *Plasmodium falciparum*, SYBR Green, Susceptibility

## Abstract

**Background:**

The recently introduced SYBR Green1 (SG) assay for testing parasites susceptibility to anti-malarial drugs needs further improvement. This has been necessitated by various setbacks, the major one being the low fluorescence intensity associated with it use. This shortcoming diminishes the anticipated hope that this novel method was going to replace the more traditional ones, such as the isotopic and microscopy. In order to restore confidence in its use, series of experiments to determine conditions that give the best fluorescence intensity were conducted.

**Methods:**

Conditions that yield the maximum fluorescent signal were ascertained by measuring the fluorescence after incubation of *Plasmodium falciparum* culture at different parasites concentration with lysis buffer containing SYBR Green (LBS) at different time period. In order to ascertain the effect of freeze–thaw on fluorescence intensity, *P. falciparum* culture was frozen for 1 h, thawed, incubated with LBS and the fluorescence measured. The optimized conditions determined in this study were then used to assess the susceptibility of clinical isolates of *P. falciparum* to artesunate, chloroquine and mefloquine. The concentration of anti-malarial drug inhibiting parasite growth by 50 % (IC_50_) for each drug was estimated using the online ICEstimator. The IC_50_ generated using the optimized SG method determined in this study was compared with that obtained using microscopic method and the previously reported standard SG method.

**Results:**

Over all, the SG method was found to be easy to perform and sensitive. Freeze–thaw of parasite culture followed by incubation with lysis buffer containing the dye for 3 h was consistently observed to give the highest fluorescence signal. The IC_50_ values for chloroquine, mefloquine and artesunate determined were consistent and comparable with that determined with the previously reported standard SG method and the microscopic method.

**Conclusion:**

The authors conclude that freezing and thawing of parasite culture, followed by incubation with LBS in the dark for 3 h provided a significant improvement in fluorescence signal. The IC_50_ generated using the improved SG method is comparable with that from microscopy and the standard method.

## Background

With the introduction of artemisinin-based combination therapy (ACT) as the first-line anti-malarial drug, continuous monitoring of parasite response to these frontline drugs is necessary. Data collected from such surveillance over a period will allow for early detection of *Plasmodium falciparum* resistance to the drug. Although the in vivo method of assessing parasites susceptibility to anti-malarial drugs remains the “gold standard” its use is limited by the running cost [1] and also the fact that studies involving the use of artemisinin monotherapy are not permissible in disease-endemic countries because of ethical issues. Molecular analysis for resistance associated with the artemisinin is not feasible because genuine markers for typical artemisinin resistance phenotype are yet to be described. Recently, Ariey et al. [2] described some markers for artemisinin resistance, but these are yet to be globally validated. In the light of these limiting factors, the in vitro drug sensitivity test has become a strong choice for assessing anti-malarial drug efficacy.

The in vitro method of assessing parasites susceptibility to anti-malarial drugs is relatively cheap and can rapidly be performed. Various methods of assessing the outcome of the in vitro drug tests have been described. These include the [^3^H] hypoxanthine uptake test [3] and the WHO micro-test [4]. However some of these methods have limitations, such as radioactive waste disposal, expensive equipment, as well as a requirement for a high level of expertise [3, 5]. Recently, a SYBR Green 1(SG)-based in vitro method was described [6–8] and reported to show promising potential to replace the traditional methods. SYBR Green is an asymmetrical cyanine dye which binds directly to double strand DNA, preferring G and C base pairs. When intercalated into DNA, it is highly fluorescent, absorbing light at a wavelength between 390 and 505 nm, with a peak at 497 nm and a secondary peak near 254 nm. It emits light at 505–615 nm, with a peak at 520 nm.

Although validated and found to be fast and relatively inexpensive, growth assessment using the SG-based method is reported to have inherent limitations such as low fluorescence reading and high background noise [9]. It was assumed that the presence of haemoglobin in the parasite culture could be responsible for quenching of fluorescence as previously reported for a similar fluorochrome [10]. However the method as reported by Johnson and colleagues assumes that the addition of the lysis buffer containing SG (LBS) is enough to cause complete disruption of the red blood cells (RBC). The group also recommends incubation with the LBS for an hour, which may not be sufficient for optimal binding of the dye. In this study, the effect on fluorescence when the incubation period of culture with LBS was extended up to 24 h at fixed time intervals was ascertained. Additionally, another procedure to ensure maximum disruption of RBC in order to increase parasite DNA and hence fluorescence was introduced by freezing and thawing of culture before plate reading. The optimal conditions discovered were used in an in vitro test of susceptibility of *P. falciparum* field isolates to chloroquine, mefloquine and artesunate.

## Methods

### Relationship between parasite DNA and SYBR Green-1 fluorescence

According to Bacon and colleagues, the SG molecule intercalates in the genomic DNA of the malaria parasite and then fluorescence [1]. In order to demonstrate this, the relationship between parasite DNA and SG fluorescence was examined. Briefly, *P. falciparum* DNA from a 10 % parasite culture was extracted using standard method. After determining the concentration of the parasite DNA it was serially diluted with DNase-free water to generate a concentration range. One hundred microlitres of each concentration was dispensed into a 96-well plate followed by the addition of 100 μl lysis buffer [20 mM Tris (pH 7.5), 5 mM EDTA, 0.008 % (W/V) saponin, and 0.08 % (V/V) Triton X-100] containing SG (1× final concentration)—here in referred to as LBS. Control wells consisting of 100 μl Dnase free water and equal volume of LBS were also set up. Plates were incubated in the dark at room temperature for 1 h and the fluorescence intensity was measured at 485 nm excitation and 528 nm emission using a BMG labtech FluoStar optima analyzer. Fluorescence units were then plotted against parasite DNA concentration. Each data point was performed in triplicate and three independent experiments were performed.

### Effect of haemoglobin on SYBR Green fluorescence

Uninfected blood was centrifuged and washed in RPMI 1640 to remove plasma and buffy coat. The pellet was diluted with culture medium to generate a haematocrit range of 0.3–5 %. Each haematocrit was spiked with parasite DNA (prepared above) at a fixed concentration (0.2 ng/µl). Hundred microlitres of each mixture was dispensed into the wells of a microtitre plate and an equal volume of LBS was added. The plate was incubated in the dark for at least 1 h and fluorescence was measured as previously described. Fluorescence readings were plotted against haematocrit and the correlation determined.

### Relationship between parasitaemia and SYBR Green1 fluorescence

One hundred microlitres of *P. falciparum* culture at an initial parasitaemia of 7.8 % was serially diluted in a 96-well microtitre plate with blood at a fixed haematocrit to yield a concentration range of 0.03–7.8 %. A direct lysis of the blood cells and staining of parasite DNA was done by the addition of 100 μl of LBS to each well. After incubating the plates in the dark for 1 h, fluorescence was measured at 485 nm (excitation) and 528 nm (emission) as usual. Parasite level was then plotted against fluorescence intensity and the correlation determined.

### Maximum time of incubation with LBS for optimum SYBR Green1 fluorescence and the effect of freeze–thaw

Incubation period of *P. falciparum* culture with LBS that yields the best fluorescent signal and the effect of freeze–thaw fluorescence intensity were ascertained. Briefly, 100 μl of *P. falciparum* culture at an initial parasitaemia of 7.8 % was serially diluted to give a concentration range of 0.03–7.8 % parasitaemia in a 96-well microtitre plate. Direct lyses of the blood cells to release the parasite DNA was done by adding 100 μl of LBS to each well. After incubating the plates in the dark for 5 min, fluorescence were measured as previously described. Thereafter, the plate was kept in the dark and fluorescence intensity determined after 1, 3 and 24 h incubation in the dark.

A second plate containing the same parasite concentration range was prepared in parallel to that described above. However, this plate was frozen immediately at −20 °C after which the culture was thawed and 100 μl LBS added. The plate was then incubated in the dark and the fluorescence read after 3 h. Fluorescence readings were plotted against parasitaemia.

### Determination of IC_50_ using optimized conditions

Blood samples were collected from children participating in an ongoing anti-malarial drug efficacy study. A total of 20 children with confirmed mono-infection of *P. falciparum* each donated 2 ml blood for the trial after their parents/guardian consent have been sought. Incomplete RPMI 1640 culture media supplemented with hypoxanthine and glucose were prepared as previously described [7]. Complete RPMI 1640 contains NaHCO3 and Albumax (Invitrogen). Chloroquine, mefloquine and artesunate used in this study were supplied by the World Wide Anti-malarial Resistance Network (WWARN).

The collected blood was diluted 20× with complete RPMI 1640 and 100 µl added to each well of a pre-dosed test plate containing chloroquine at a concentration range of 7.8–2000 ng/ml; mefloquine 1.9–500 ng/ml; and artesunate 0.78–200 ng/ml. Wells containing no drug but diluted patient’s blood was included on each plate as control. A culture of laboratory reference clone, 3D7, regarded as chloroquine sensitive at an initial parasitaemia of 0.5 % and haematocrit of 1.5 % was also tested in parallel as additional control. The plates were placed in a modular incubator chamber and gassed (gas contains 92.5 % N_2_, 5.5 % CO_2_, 2 % O_2_). The chamber containing the culture was placed in an incubator at 37 °C for 72 h. The assay was terminated by freezing the plate at −20 °C for at least 1 h before thawing. Hundred microlitres of LBS was added to each well and mixed thoroughly by gently tapping on the plate. The plate was covered with aluminum foil and incubated at room temperature in the dark for 3 h. Fluorescence was then read as usual. A second plate using the same patient sample and drugs was set up in parallel. Parasite growth in the second plate was monitored in the drug free well from 20 h post-plating, by preparing blood smears using the cells in the drug free-wells. Once 60 % of the parasites in the drug-free well have developed into schizonts the cells from each well were harvested onto a microscope slide. The smears were air dried, stained with 10 % Giemsa for 30 min and examined with the microscope under oil immersion. The *P. falciparum* schizonts in each smear were counted against 200 leucocytes. The concentration of anti-malarial drug inhibiting parasite growth by 50 % (IC_50_) for each drug was estimated from a dose response curve by non-linear regression analysis using an online program, previously described by the groups of Le Nagard and Kaddouri [11, 12]. For the SG assay fluorescence intensity was plotted against drug concentration, parasite count was plotted against drug concentration in the microscopy assay. The IC_50_ values determined using the new conditions was compared to the geometric mean IC_50_ values obtained with the same samples but assayed using the reported standard SG method. The IC_50_ values obtained with the optimized SG was also compared with those obtained with the microscopic method.

### Statistical analysis

The degree of difference between the previous conditions and that found in the current study were determined. GenStart 9th edition statistical package was used for statistical analysis. Microsoft office excels 2007 and Graphpad prism software package were also used for graphical presentation of data.

### Ethical approval

Permission to carry out this work and ethical clearance were obtained from the Institutional Review Board (IRB) of the Noguchi Memorial Institute for Medical Research (NMIMR), Ghana.

## Results

The relationship between the levels of parasitaemia and the fluorescence units determined after incubation with LBS in the dark showed a well-correlated linear relationship with r^2^ = 0.9962. Similar trend was observed in a plot of parasite DNA concentration against fluorescence intensity. SYBR Green fluorescence signal was found to decreases with increasing haematocrit levels (R^2^ = 0.9492).

**Fig. 1:**
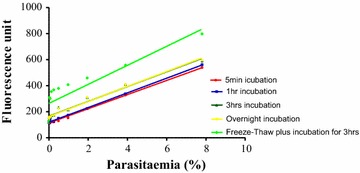
Effect of length of incubation with LBS and freeze–thaw on intensity of fluorescence signal. Fluorescence was measured at 485 nm excitation and 528 nm emission

It was observed in this study that freeze–thaw followed by incubation with SG for 3 h gave the highest fluorescence intensity (Fig. 1). The results showed a well-correlated linear relationship (r^2^ = 0.9962) for freezing the samples for 1 h followed by thawing before the addition of LBS. Though there was a significant increase in fluorescence signal when incubation time was increased to 3 h, there appear to be not much gain in fluorescence when the incubation time was extended overnight. The difference between fluorescence signal for freezing samples for 1 h followed by a 3-hour incubation with LBS and incubating the samples 1 h after the addition of LBS without freezing was significant (F = 3.42 and P = 0.078). This trend was consistently observed in 3 independent experiments.

**Fig. 2:**
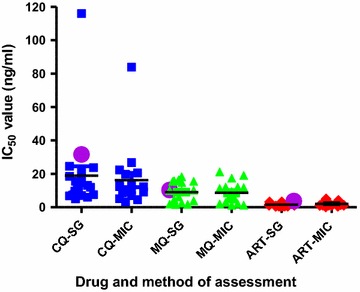
Scatter plots of IC_50_ values from assessment of the susceptibilities of *P. falciparum* clinical isolates to chloroquine *CQ*, mefloquine *MQ* and artesunate *AS* generated using the optimized SG method and the microscopic method. The IC_50_ values obtained using the two methods were ran side by side and designated CQ-SG, MQ-SG and AS-SG for values obtained with the SG method. For those generated with the microscopic method they were designated CQ-MIC, MQ-MIC and AS-MIC. The *purple closed circles* in the plot represent the geometric mean IC_50_ values determine with the standard SG method. NB:* CQ* chloroquine,* MQ* mefloquine,* AS* artesunate,* SG* SYBR Green method and* MIC* microscopic method

**Fig. 3:**
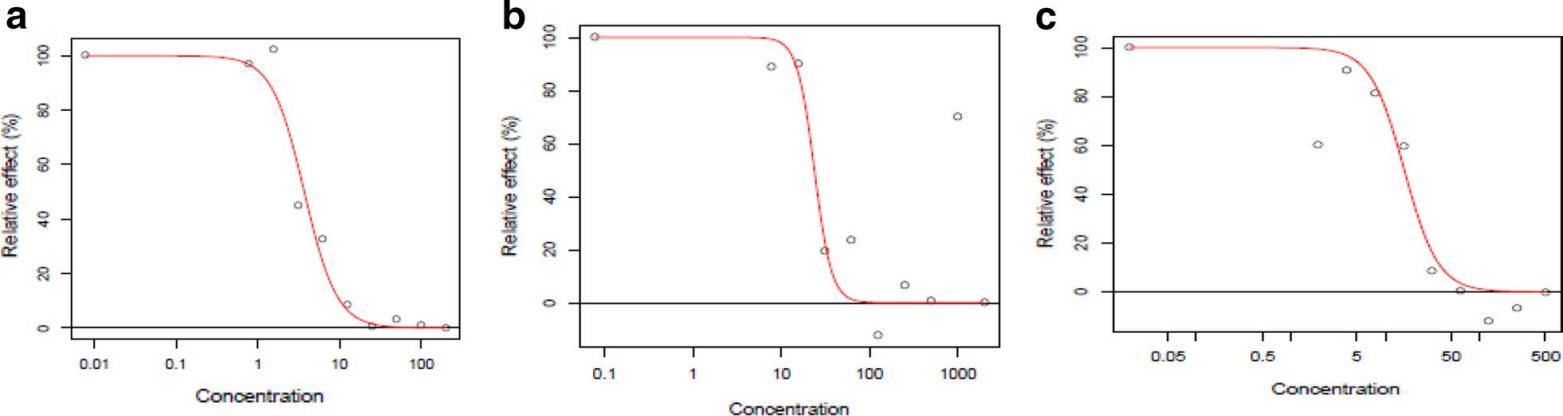
Representative *sigmoidal graphs* showing the response of *P. falciparum* clinical isolates to artesunate (**a**), chloroquine (**b**) and mefloquine (**c**). These were obtained using the optimised SG method being reported here

The outcome of the in vitro test of susceptibilities of 20 *P. falciparum* clinical isolates to CQ, MQ and AS generated using the optimized conditions determined in this study is showed in Fig. 2. The IC_50_ values obtained were within normal range and similar to that obtained with the previously reported standard SG method. The IC_50_ for each drug generated using the same patient sample and run in parallel under similar condition but assessed using the microscopy method is also plotted alongside in Fig. 2. The IC_50_ generated for each drug using the different methods were found to be comparable. Figure 3 shows typical sigmoidal graphs from the in vitro drug test using the current optimized SG method.

## Discussion

Further optimization of the recently introduced SG method for in vitro drug test was warranted because of some challenges, particularly low fluorescence signal, associated with it use. Some of the critical stages in running of the assay were systematically re-examined in order to determine the best fluorescence intensity.

Consistently, it was observed that freezing and thawing, followed by incubation with LBS in the dark for 3 h gave the highest fluorescence signal. Earlier, Johnson et al. reported that freezing the plates before or after the addition of LBS didn’t lead to significant increase in fluorescence signal [7]. Contrary, Smilkstein et al. showed a significant effect of freeze–thaw on fluorescence [6]. Evidence provided in this study settles this controversy. It appears freezing the culture for at least an hour, followed by thawing ensures complete lysis of the RBC leading to an increased availability of parasite DNA for staining. This obviously led to an increase in fluorescence intensity. Although incubation of LBS with culture for 1 h at room temp in the dark was reported to give a readable fluorescence by Johnson et al. [7], a marked increase in fluorescence signal when the period was extended to 3 h was observed in this study (F = 1.35 and P = 0.257). However, not much difference in fluorescence was seen after an overnight incubation. It could therefore be said that that 3 h is sufficient time for a complete interaction between the SG dye and the parasite DNA. These findings are important as it suggest that carrying delicate equipment to the field in order to perform the assay is not necessary. The test plates can be frozen and later transported to the main laboratory for fluorescence reading.

A decreasing trend in fluorescence with increasing haematocrit at fixed parasite DNA concentration observed in this study was expected. This may be attributed directly to the quenching effect of the red blood cells as previously reported [13]. This phenomenon could probably be due to the fact that the absorption spectrum of red blood cell overlaps with the excitation and emission wavelength of SG at 485 nm and 529 nm. Though SG has high affinity for DNA, its inability to indiscriminate detection of parasitical and non-parasitical DNA resulting in high background noise has affected the initial enthusiasm the method enjoyed among scientist. This situation could limit the application of the method for field drug sensitivity testing. However increasing the fluorescence signal using the optimized condition could counter this phenomenon and restore confidence in its use. The enhance fluorescence also means samples with very low parasitaemia could easily be assayed. It must however be re-emphasized that the assay should be performed with the minimum amount of RBC possible.

Comparison between the isotopic and the SG method had earlier been performed by Johnson et al. [7]. A close resemblance of the two methods was reported. It is however worth mentioning that the isotopic method is associated with the acquisition and disposal of harmful radioactive waste and it is far more expensive to perform compared to the SG method. For these and other operational reasons the SG method is preferable.

The IC_50_ values obtained using the optimized conditions appear to be within the expected range and comparable to the IC_50_ values obtained using the microscopy method. However, it was noted that, comparatively, the microscopic was tedious to perform and prone to human errors. Earlier, Quashie et al. reported overall geometric mean IC_50_ values of 3.8, 31.56 and 10.12 ng/ml for artesunate, chloroquine and mefloquine respectively using the standard SG method [14]. Since samples used in this study are a sub-population of the aforementioned study the IC_50_ values were compared side by side and observed to be similar or identical. However, the significant increase in fluorescence with the optimized method would increase accuracy and sensitivity of the measurements. Indeed it will permit assessment of samples with very low parasitaemia and also ensures confidence in the use of the method.

All put together, evidence obtained in this study gives further credence to the fact that the SG method is consistent and could replace the traditional methods for the routine assessment of parasites sensitivity to drugs more so with the enhanced signal intensity herein reported. Data obtained from continuous anti-malarial drug monitoring system will ensure early detection of the emergence of parasite strains with reduced sensitivity to the newly introduced artemisinin drugs.

## Conclusion

From observations made in this study, it could be conclude that freezing and thawing of culture, followed by incubation with LBS in the dark for 3 h gives the highest fluorescence signal. This information is important for attaining the full benefit of using the recently introduced SG method in drug monitoring system.
